# Endograft position and endoleak detection after endovascular abdominal aortic repair with low-field tiltable MRI: a feasibility study

**DOI:** 10.1186/s41747-023-00395-0

**Published:** 2023-12-21

**Authors:** Jordy K. van Zandwijk, Richte C. L. Schuurmann, Bennie ten Haken, Chrit M. Stassen, Robert H. Geelkerken, Jean-Paul P. M. de Vries, Frank F. J. Simonis

**Affiliations:** 1 Magnetic Detection & Imaging, Technical Medical Centre, University of Twente, Enschede, The Netherlands; 2 Department of Vascular Surgery, Medisch Spectrum Twente, Enschede, The Netherlands; 3 Department of Surgery, Division of Vascular Surgery, University Medical Center Groningen, Groningen, The Netherlands; 4 Multimodality Medical Imaging Group, Technical Medical Centre, University of Twente, Enschede, The Netherlands; 5 Department of Radiology, Ziekenhuisgroep Twente, Hengelo, The Netherlands

**Keywords:** Aortic aneurysm (abdominal), Endoleak, Magnetic resonance imaging, Standing position, Supine position

## Abstract

**Background:**

Abdominal aortic endoleaks after endovascular aneurysm repair might be position-dependent, therefore undetectable using supine imaging. We aimed to determine the feasibility and benefit of using a low-field tiltable magnetic resonance imaging (MRI) scanner allowing to study patients who can be imaged in both supine and upright positions of endoleaks.

**Methods:**

Ten EVAR patients suspected of endoleak based on ultrasound examination were prospectively included. MRI in upright and supine positions was compared with routine supine computed tomography angiography (CTA). Analysis was performed through (1) subjective image quality assessment by three observers, (2) landmark registration between MRI and CTA scans, (3) Euclidean distances between renal and endograft landmarks, and (4) evaluation of endoleak detection on MRI by a consensus panel. Statistical analysis was performed by one-way repeated measures analysis of variance.

**Results:**

The image quality of upright/supine MRI was inferior compared to CTA. Median differences in both renal and endograft landmarks were approximately 6–7 mm between upright and supine MRI and 5–6 mm between supine MRI and CTA. In the proximal sealing zone of the endograft, no differences were found among all three scan types (*p* = 0.264). Endoleak detection showed agreement between MRI and CTA in 50% of the cases, with potential added value in only one patient.

**Conclusions:**

The benefit of low-field upright MRI for endoleak detection was limited. While MRI assessment was non-inferior to standard CTA in detecting endoleaks in selected cases, improved hardware and sequences are needed to explore the potential of upright MRI in patients with endoleaks.

**Relevance statement:**

Upright low-field MRI has limited clinical value in detecting position-dependent endoleaks; improvements are required to fulfil its potential as a complementary modality in this clinical setting.

**Key points:**

• Upright MRI shows potential for imaging endoleaks in aortic aneurysm patients in different positions.

• The image quality of upright MRI is inferior to current techniques.

• Upright MRI complements CTA, but lacks accurate deformation measurements for clinical use.

• Advancements in hardware and imaging sequences are needed to fully utilise upright MRI capabilities.

**Graphical Abstract:**

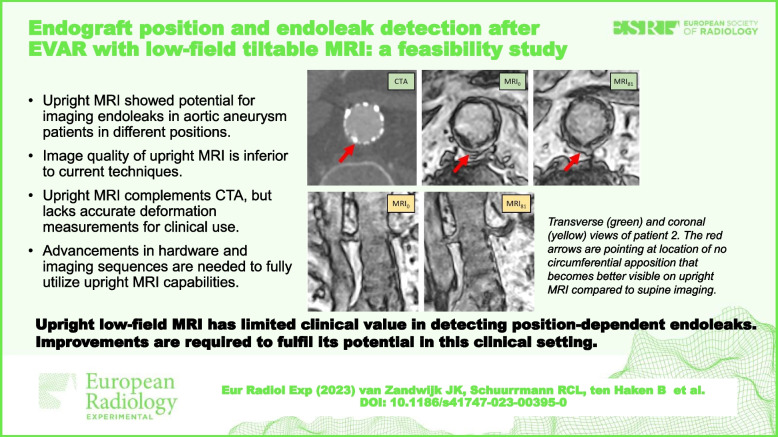

## Background

Endovascular aneurysm repair (EVAR) is a minimally invasive technique to repair abdominal aortic aneurysms. Follow-up after EVAR is done for detection of complications such as endoleaks with colour Doppler ultrasound (CDUS) or contrast-enhanced computed tomography angiography (CTA) [1]. International guidelines advise triple-phase CTA within 30 days after EVAR, followed by annual CDUS monitoring when no complications are suspected [2, 3]. CTA is considered the standard of practice with a sensitivity as high as 92% in detecting endoleaks and other endograft-related complications [4]. Conventional radiography is used as a supplementary modality to CDUS to detect significant migration or stent fractures implying a lower radiation exposure than CTA [4]. Magnetic resonance angiography (MRA) has been suggested in selected patients with iodinated contrast allergy, at risk for contrast-induced nephrotoxicity, or inconclusive imaging findings on other modalities [5]. Additional value of MRA is also found in using non-contrast-enhanced techniques which completely eliminate the use of potentially toxic contrast agents and the ability of aneurysmal sac content characterisation [6].

In more than 40% of EVAR cases, the aneurysm shows no sac shrinkage during follow-up, which could indicate that the aneurysmal sac is still perfused due to an endoleak [7]. Type V endoleaks are defined as aneurysm growth on CDUS or CTA without visible endoleak on appropriate imaging techniques. This type of endoleak is referred to as endotension and has a reported incidence of 1–5% [8]. It has been postulated that these endoleaks may be position-dependent [9, 10]. Previous studies have reported cases of EVAR patients with demonstrated position-dependent endoleaks [10–12]. Specific body movements, such as changes in position or flexing the spine, can influence the position and deformation characteristics of the aorta in the body. Moreover, the upright position of the human body adds gravitational dependence on the aorta, endograft, and aneurysmal sac, which can also affect their positions [11]. Consequently, standard supine follow-up imaging after EVAR may miss endoleaks that occur only in specific positions or body postures [13]. While type V endoleaks are of interest due to their undefined causes, other types of endoleaks, such as Ia, also merit attention as potential position-dependent cases where the leakage may worsen with different body positions [12]. These considerations lead to the question of whether position-dependent imaging of EVAR patients suspected of having an endoleak with no clearly identified cause would provide added value.

Magnetic resonance imaging (MRI) with a tiltable scanner provides an opportunity to scan patients in both supine and upright positions and therefore may be able to detect position-dependent endoleaks. Also, the acquisition of three-dimensional volumetric data in upright and supine positions can increase insights into changes in aortic and endograft deformation when changing body position. Moreover, as open MRI scanners generally operate at lower magnetic field strengths (< 1.0 T) compared to conventional MRI, the potential additional artifacts arising from the metal components of endografts are further reduced [14]. This reduction in artifacts can potentially enhance imaging around the endografts.

The aim of this study is to investigate the feasibility and added value of upright low-field MRI to clarify the characteristics of type V endoleaks. Therefore, the objectives of this study were (i) to evaluate the image quality of supine and upright MRI focusing on endograft and aorta landmarks, (ii) to analyse whether changes in the deformation of the aorta and endograft between supine and upright positions could be identified, (iii) to assess differences in the proximal sealing zone of the endograft between upright and supine MRI examinations, and (iv) to subjectively assess the detectability of endoleaks using either supine or upright MRI. A comparison was made with CTA as a reference standard to investigate whether upright low-field MRI could provide useful information or even reveal details not visible on CTA.

## Methods

### Study design and population

Between January 2020 and August 2021, ten patients with an abdominal aortic aneurysm who were treated with EVAR were recruited from the Department of Vascular Surgery of Medisch Spectrum Twente, Enschede, the Netherlands. This prospective study was approved by the regional Medical Ethical Committee (NL69413.091.19). All patients gave written informed consent.

To be eligible, the patients had to meet the inclusion criteria: (1) they must have had a previous EVAR intervention with either an Endurant (Medtronic, Minneapolis, USA) or Anaconda (Terumo Aortic, Inchinnan, Scotland, UK) endoprosthesis and (2) the patients had to undergo a CTA scan due to detected growth of the aneurysm or a suspected endoleak (of all types) based on CDUS examination. Patients were excluded if they met one or more of the following exclusion criteria: (1) abdominal waist of more than 47 cm (left–right) or 29 cm (anterior–posterior) determined on CTA (because of coil restrictions); (2) negative result from the MRI safety checklist; (3) inability to stand for 15 min without assistance; and (4) outdated CTA scan (> 2 months before MRI examination).

### MRI acquisition

Images were acquired using a 0.25-T scanner (G-scan; Esaote, Genoa, Italy) in the supine (0° rotation with respect to horizontal plane = MRI_0_) and the upright (81° = MRI_81_) position. The standing position of 81° was chosen such that patients would not fall over and could more easily remain stable during examination than in a 90° position. The MRI parameters in the imaging protocols were set with maximal focus on deformation properties that characterise the endograft and the aortic wall. First, a quick scan (localiser) was made to correctly position the patient with lumbar vertebra L2 in the isocentre of the magnet. Second, a scan protocol was performed (parameters are displayed in Table 1) consisting of transverse and coronal balanced steady-state free precession and spoiled gradient echo sequences. The total scan time of all sequences in supine and upright positions including pre-scan calibration was approximately 30 min. Patients were first scanned in an upright position, followed by supine scanning to prevent fainting caused by orthostatic hypotension [15].

**Table 1:** Technical parameters of MRI sequences

	Name	Ori	TE	TR	FA	NSA	FOV	F × P × S	IR	AT
1	3D bSSFP	Cor	4	8	40	3	270 × 270 × 84	172 × 172 × 48	0.53	03:57
2	3D SGE	Tra	14	30	35	1	280 × 280 × 104	212 × 212 × 58	0.55	04:49
3	3D bSSFP	Tra	4	8	40	2	270 × 270 × 84	244 × 244 × 58	1.05	05:33
4	3D SGE	Cor	14	30	30	1	270 × 270 × 105	172 × 188 × 48	1.06	04:53

*AT* Acquisition time (min:s), *FA* Flip angle (degrees), *FOV* Field of view (mm × mm × mm), *IR* Isotropic resolution (mm), *NSA* Number of signal averages, *ORI* Orientation, *TE* Echo time (ms), *TR* Repetition time (ms), *F* × *P* × *S* Frequency × phase × slice phase

CTA scans were acquired on a dual-source CT scanner (Somatom Definition Flash; Siemens Healthineers, Forchheim, Germany) using a standardised electrocardiographically gated protocol. This protocol involved the following acquisition parameters: dynamic tube voltage between 80 and 120 kV with an automated modulating tube current, pitch depending on actual heart rate, rotation time 0.33 s, collimation 128 × 0.6 mm. The data was reconstructed using reconstruction kernel I26f to slices with 1-mm thickness and 0.5-mm spacing between slice locations, field of view 400 × 400 mm^2^, matrix size 512 × 512, resulting in voxels of 0.78 × 0.78 × 1 mm^3^. A total amount of 80 cc of iodinated contrast agent (Optiray 300, Guerbet LLC, Princeton, USA) was used, injected with a flow rate of 4 mL/s. Multiphase acquisition was performed, producing pre-contrast, arterial, and delayed phases.

### Image quality analysis

In accordance with the first objective regarding image quality, acquired MRI in upright and supine positions were independently analysed by a technical physician (R.S.), a radiologist (C.S.), and a vascular surgeon (R.G.), each of them with over ten years of clinical experience in assessment of vascular images. The three-dimensional transverse spoiled gradient-echo series was used as the main volume in a multiplanar reformatted view to assess whether the following landmarks could accurately be identified: (i) distal border of the left renal artery (LRA); (ii) distal border of the right renal artery (RRA); (iii) most superior point of the endograft (E-S) visible when viewing perpendicular on the aorta (*i.e.*, bare stent for the Endurant, saddle rings for the Anaconda); (iv) proximal border of the endograft fabric (E–F) (Endurant only); and (v) the circumferential apposition of the endograft with the aorta (E-A). Balanced steady-state free precession sequences could additionally be consulted for improved detection of the renal arteries in more complex cases. A 4-point Likert scale was used, with a subjective distinction between not assessable, poorly assessable, sufficiently assessable, and good assessable points.

**Fig. 1:**
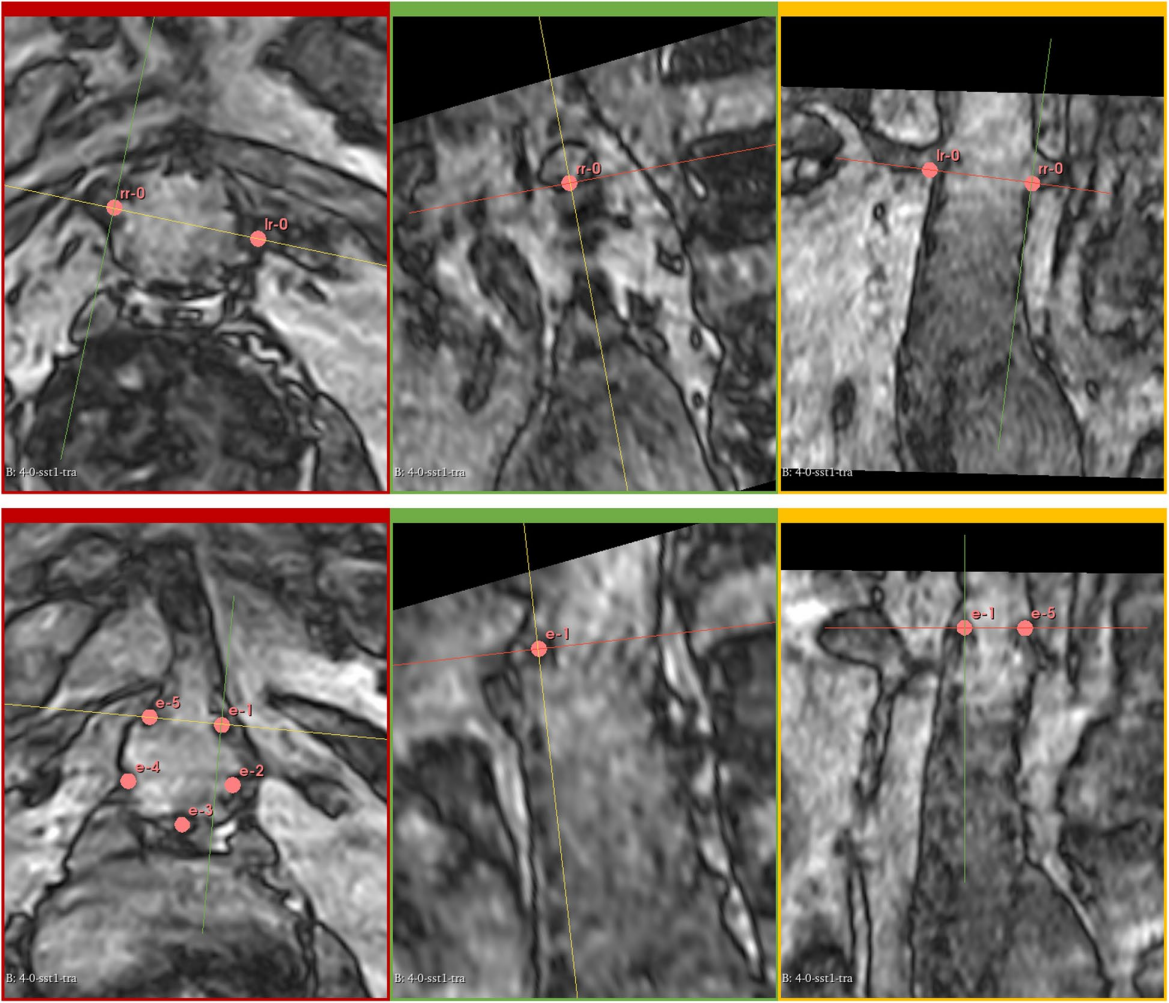
Landmark placement of the renal arteries (top row) and endograft bare stent markers (bottom row) in patient 2. Transverse (red), sagittal (green), and coronal (yellow) views were reformatted such that all markers are visible in the same view

For a mutual comparison between MRI_81_ and MRI_0_ scans, the data were exported to 3D Slicer (slicer.org, Ver 5.0.3) [16]. On the MRI_81_, MRI_0_, and CTA scans, landmarks were placed at the origins of the LRA, the RRA, and at the most proximal points of the implanted endograft (see Fig. 1). For the Endurant endograft, these are five points (E1-E5) on the tips of the bare stent, and for the Anaconda endograft these are four points (E1-E4) on the two peaks and on the two valleys of the most proximal saddle-shaped ring. Since there was no known experience in determining anatomical and endograft landmarks on low-field MRI data in this population, the positioning of landmarks was simultaneously and jointly performed and fine-tuned in a consensus meeting by four authors (J.V.Z., F.S., R.S., and R.G.) to ensure optimal analysis of the data. MRI_81_, MRI_0_, and CTA data were randomly assessed to prevent prior knowledge of the cases to bias the placement of the landmarks. Moreover, at least two months elapsed following the initial image quality assessment by all three observers to minimise the potential recall bias. By means of internal discussion, the most optimal landmark placements were determined. No landmarks were placed when the desired landmark could not be detected on the scan by the consensus panel.

### Aorta and endograft deformation

To answer the second objective regarding the deformation of the aorta and endograft, a semiautomatic registration was used and applied on MRI_81_ and CTA data to determine a transformation towards the MRI_0_ frame. This reference frame was chosen to analyse differences with the reference standard CTA in MRI_0_-CTA and between supine and upright MRI in MRI_0_-MRI_81_. Through the registration of the different scans with each other, the landmark positioning and thus differences in aorta and endograft deformation could be determined. The landmark registration module in the 3D Slicer software was used to register both scans towards the coordinate system of the MRI_0_ scans with at least two landmarks on the most anterior point of the vertebrae L2 and L3. Refinement of the transformation was performed by visual inspection of overlapping scans, focusing on the best overlap of the vertebral column. Similarly, the CTA was registered to the MRI_0_ frame to allow for validation of the analysis. The transformations between MRI_81_ and MRI_0_ data and CTA and MRI_0_ were applied to the landmark coordinates that were placed as described above. Euclidean distances were calculated between the coordinates of both renal landmarks and all endograft landmarks, and the results were described using median values with interquartile ranges across all patients.

### Position-dependent proximal sealing zone

Two centres of gravity were calculated, one between both renal arteries and the other among all endograft markers in all scans, to assess differences in the proximal sealing zone of the endograft. This was the third objective of this study. Similarly as with the aorta and endograft deformation, Euclidean distances were calculated between both centres of gravity, and the results were described using median values with interquartile ranges across all patients.

### Endoleak detection

In accordance with the fourth objective of this study, MRI clinical findings were subjectively made and categorised into additional findings compared to the CTA (noted as +), agreement on MRA with CTA (noted as =), or no agreement with the CTA findings (noted as −). All assessments were made by our consensus panel and verified by a radiologist with 25 years of experience in assessing vascular images (C.S.) Additional findings on MRA were defined as having a potential clinical added value in comparison to the CTA scans. Agreement of the MRI findings with CTA was noted when the most important findings on CTA radiology report causing aneurysm growth could also be seen on MRI. Similarly, no agreement would mean that CTA findings could not be verified with MRI. All findings and observations by the consensus panel, as well as those made by the radiologist afterward, were conducted in a blind and randomised order to prevent memorisation and recognition of previous scans.

### Statistical analysis

Descriptive statistics were used to present the data for the image quality part. Distances in the proximal sealing zone of the endograft, measured for all three scan types (CT, MRI_0_, and MRI_81_), were compared using a one-way repeated measures analysis of variance.

## Results

Demographics of the patients are given in Table 2. A total of ten patients (8 males, 2 females) with a median age of 78 years (interquartile range 70–81) underwent MRI after they had a multiphase contrast-enhanced CTA because of aneurysm growth or endoleak suspicion on CDUS. The time elapsed between MRI examination and CTA for all patients ranged from one week to two months. Both endoprosthesis platforms (Endurant and Anaconda) were included five times.

**Table 2:** Demographic of patients who participated in this study

Patients (*n*)	10
Height, cm (median, IQR)	179 (176–181)
Weight, kg (median, IQR)	82 (80–85)
BMI, kg/m^2^ (median, IQR)	25.9 (24.4–26.6)

### Image quality

**Fig. 2:**
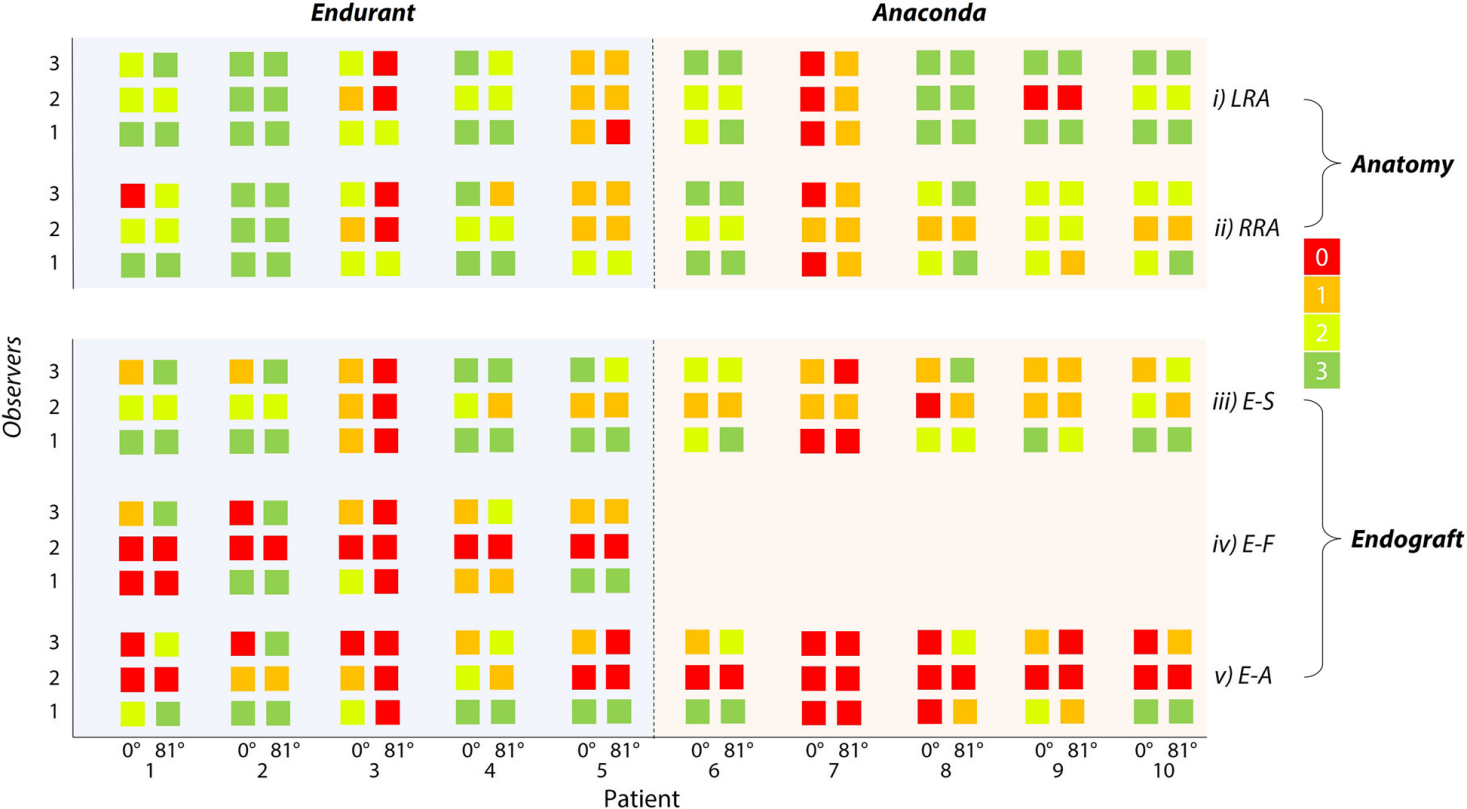
Subjective assessment by three observers in all scanned patients. *E-A*, Endograft apposition; *E–F*, Endograft fabric; *E-S*, Endograft superior point; *LRA*, Left renal artery; *RRA*, Right renal artery

Subjective assessment of the overall image by all observers revealed an inferior image quality of both MRI_0_ and MRI_81_ compared to the CTA datasets. Figure 2 shows the scores given by the three observers. On CTA, all points were good assessable. Out of 60 observations on anatomy in the supine position, there were 22 (36.7%) good scores, 21 (35%) were considered sufficiently assessable, 10 (16.7%) were poorly assessable, and 7 (11.7%) were deemed not assessable. For the upright position, these absolute values and percentages were 25 (41.7%), 15 (25%), 14 (23.3%), and 6 (10%), respectively. In one patient, there was a good score for all anatomical observations by all observers, and for the assessment of the endograft the maximum score of good assessable was given in 9 (50%) instances in this patient. For two patients with an Anaconda endograft, image quality was insufficient to place any endograft marker. In these cases, the consensus panel found that the level of uncertainty in placing the endograft markers was too high. These were omitted from the endograft analysis. For one patient, only two out of five endograft markers were sufficiently visible to be placed in the consensus meeting. Patients with the Endurant endograft were equally assessed in terms of anatomy with an accumulated score (with a maximum of 180) of 118 (65.6%) by all observers, compared to 119 (66.1%) for the Anaconda. Moreover, patients with the Endurant endograft performed better on endograft assessments in the two categories that were assessable for both endografts (iii and v), achieving an accumulated score of 99 (55%) compared to 67 (37.2%) for the Anaconda. The proximal border of the endograft fabric (category iv) was only assessable in the Endurant endograft, with an accumulated score of 29 (32.2%).

### Aorta and endograft deformation

**Fig. 3:**
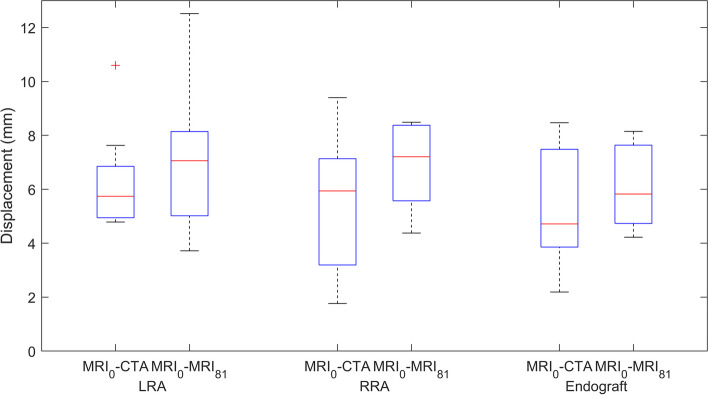
Boxplot showing left renal artery (LRA), right renal artery (RRA), and endograft landmark differences between supine MRI (MRI_0_) *versus* CTA and supine MRI *versus* upright MR (MRI_81_) scans. *CTA*, Computed tomography angiography; *MRI*, Magnetic resonance imaging

In accordance with the second objective to analyse whether changes in deformation between patients in supine and upright positions could be identified, differences between the landmarks of the renal arteries and endograft in these two positions were assessed. The median (interquartile range) difference between MRI_0_ and CTA landmarks was 5.7 mm (4.9–6.9) for the LRA and 5.9 mm (3.2–7.1) for the RRA (Fig. 3). Between MRI_0_ and MRI_81_, these differences were 7.1 mm (5.0–8.1) and 7.2 mm (5.6–8.4) respectively. Based on the distribution of distances in each direction for both renal arteries in every patient, no specific direction of displacement could be identified. For the endograft markers, the differences in position for MRI_0_ and CTA were 4.2 mm (3.3–5.7) for Endurant and 7.6 mm (4.9–8.3) for the Anaconda endografts. Between MRI_0_ and MRI_81_, this was 4.9 mm (4.4–6.6) and 7.3 mm (6.2–7.6) for the patients with respectively Endurant and Anaconda endografts.

### Position-dependent proximal sealing zone

**Fig. 4:**
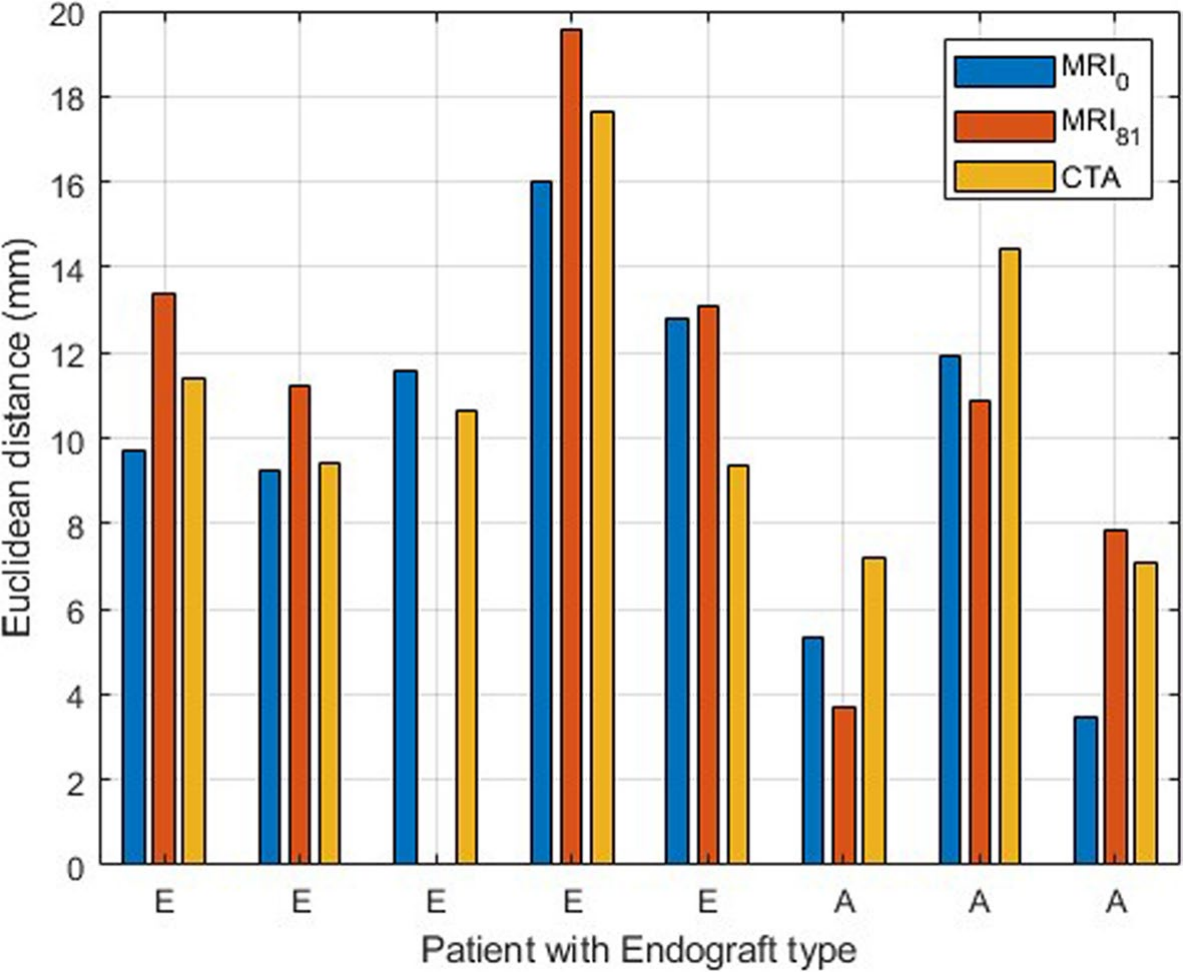
Euclidean distances between the centres of gravity of the renal and endograft for supine MRI (MRI_0_), upright MRI (MRI_81_), and CTA. Patients are categorised on the basis of their implanted endograft, either Endurant (E) or Anaconda (A). *CTA*, Computed tomography angiography; *MRI*, Magnetic resonance imaging

Figure 4 shows the differences between the centres of gravity of all renal and endograft markers in the same scan of the eight patients with sufficient image quality to place endograft markers. The data shown in this graph are a measure of the centres of gravity between renal and endograft markers and represent the differences that occur in the proximal sealing zone of the endograft. The measurements in CTA are considered the baseline with a median and interquartile range of 10.0 (8.3–12.9) mm. For the MRI_0_ and MRI_81_ scans, these were 10.7 (7.3–12.3) mm and 11.0 (5.8–13.2) mm respectively. No statistical differences between all three groups were found (*p* = 0.264). Two patients are missing because no endograft markers could be placed due to insufficient scan quality during the consensus meeting. For a third patient, the endograft markers on the MRI_81_ data were not possible to indicate.

### Endoleak detection

**Fig. 5:**
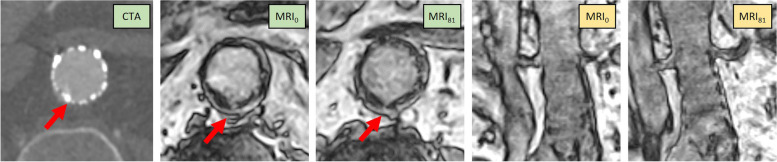
Transverse (green) and coronal (yellow) views of patient 2. The red arrows are pointing at a location of no circumferential apposition that becomes better visible on upright MRI compared to supine imaging. *MRI*, Magnetic resonance imaging

An overview of MRI clinical findings in the supine and upright positions of each patient is shown in Table 3. For the MRI_0_ scans, there were additional findings on top of the initial CTA report in 2 out of 10 cases. A reduced dorsal apposition (Fig. 5) that was initially not described by CTA was found in upright and supine MRI (patient 2) or in the supine position only (patient 3). For patient 2, a new suspicion of a type Ia endoleak arose based on reduced apposition between a lumbar artery and proximal side of the fabric. In patient 3, there was a reduced apposition in the aneurysm neck seen on MRI_0_ which was initially not noted in the radiology report. After a radiologist reviewed the CTA of patient 3, the reduced apposition could also be observed on CTA. If this reduced apposition extends up into the entire aneurysm neck, this would indicate a type Ia endoleak. This is in contrast with the type II endoleak that was noted in the initial CTA report. Agreement of MRI with the clinical CTA findings was reached in 50% of the supine and upright cases together. In two patients, agreement with CTA was only found in either supine (patient 8) or upright (patient 10) position. Only in patient 9, a type II endoleak that was visible as flow within the aneurysm on CTA was absent on both MRI scans. Contrary to CTA scans, with tiltable MRI, no direct observations of an endoleak could be made. Therefore, the notation of ‘no endoleak’ in line with CTA findings indicates that there is no causative factor for an endoleak.

**Table 3:** Results overview

					MRI findings	
pt	**Type (E/A)**	**Age (years)**	**Reason for CTA**	**CTA findings**	**Supine (**MR_0_**)**	**Upright (**MR_81_**)**
1	E	0.3	Status after EVAR, possible endoleak seen on CDUS	No endoleak and unchanged aneurysm dimensions	No endoleak ( =)	No endoleak ( =)
2	E	11.8	Growth on CDUS follow-up, type II suspicion	Type II endoleak from a right LA	Right LA visible ( =), between LA and proximal side of fabric less apposition ( +), type Ia endoleak	Right LA visible ( =), between LA and proximal side of fabric less apposition ( +), type Ia endoleak
3	E	4.4	Status after EVAR and type II embolisation from IMA, growth and flow outside prosthesis on CDUS	Aneurysm sac growth by type II endoleak after two prior embolisations	Type II outside FOV ( −), less apposition dorsal left ( +)	*Not assessable* ( −)
4	E	2.1	Growth on CDUS, possible endoleak	Large type II endoleak from IMA and an LA	Both endoleaks from IMA and LA outside FOV, no flow visible in sac ( −)	Both endoleaks from IMA and LA outside FOV, no flow visible in sac ( −)
5	E	1.1	Follow-up after EVAR, known occlusion AFS right, aneurysm growth (pseudoaneurysm)	Unchanged diameters aneurysm sac, no indication for endoleak	No endoleak ( =)	No endoleak ( =)
6	A	1.6	Growth on CDUS, possible type Ia endoleak	Type II endoleak from two LAs	Both LAs visible ( =)	Both LAs visible ( =)
7	A	2.6	Growth on CDUS with known endoleak, possible type II	Growth by type II endoleak	*Not assessable* ( −)	*Not assessable* ( −)
8	A	9.0	Status after embolisation of two LAs, now growth on non-contrast CTA	Unchanged aneurysm sac dimensions, persistent type II endoleak from an LA	Left LA connection with sac visible ( =)	No LA visible ( −)
9	A	5.3	Growth on CDUS with flow in aneurysm	Type II endoleak from an LA	No LA visible ( −)	No LA visible ( −)
10	A	8.4	After prior LA embolisations, still growth on CDUS	Aneurysm growth based on endoleak type II from an LA	No LA visible ( −)	LA visible ( =)

Observations for both MRI_0_ and MRI_81_ in relation to the CTA findings are reported in the last column. Agreement of MRI findings with CTA is depicted by ( =), whereas agreement plus additional findings is noted with ( +) and no agreement with ( −). *FOV* Field of view, *IMA* Inferior mesenteric artery, *LA* Lumbar artery

## Discussion

This study investigated the feasibility and added value of upright MRI in EVAR patients. The first objective was to evaluate image quality; both MRI_0_ and MRI_81_ had inferior image quality compared to the gold standard, CTA, in terms of contrast and spatial resolution. Although these results were expected, given the higher spatial resolution of CTA compared to MRI, the aim of all objectives of this study was to illustrate where and how CTA outperforms MRI. The addition of MRI contrast may increase the detectability of the landmarks and endoleak, but at the research location of our MRI scanner, injection of contrast agents is strictly prohibited. Based on the results of three experienced observers, the image quality in the patients with both Endurant and Anaconda endografts was sufficient in four out of five patients to continue with clinical analysis and interpretation. However, in the studied population of ten patients, there were no detectable changes in the deformation of the aorta and endograft between supine and upright positions, which answered our second objective. We discovered that upright MRI was unable to detect small differences (< 6 mm) in the aorta or endograft position in relation to supine imaging. Additionally, for our third objective regarding differences in the proximal sealing zone of the endograft, no differences were observed in the Euclidean distances between renal-endograft measurements between upright and supine positions using MRI, as well as between supine MRI and CTA examinations. For the fourth objective on the subjective assessment of endoleaks, confirmation of CT-verified type II endoleak with the currently used MRI settings without the use of contrast agent was possible in 50% of the cases with both MRI_0_ and MRI_81_. In one case, there were additional findings on both MRI_0_ and MRI_81_ that are potentially interesting and would require further clinical monitoring.

Displacement of the aorta based on different body positions has been studied before. Qian et al. studied the supine *versus* prone position with MRI but did not find any significant changes in a low thoracic-high lumbar region [17]. Similarly, at higher levels of the thoracic aorta (T4-T12), a significant relationship between patient positioning and the aorta’s relative position was demonstrated between supine and prone positions [18]. This was also found by Jiang et al., who concluded that the aorta shifts more anteromedially and closer to the spine by ~ 4 mm at T5–T10 levels when patients are changed from the supine to the prone position [19]. This indicates that the aorta or entire aorta-stent complex displaces at higher levels, although this was not confirmed in our study at the level of the renal arteries (L1-L2). However, our analysis of the position-dependent proximal sealing zone does provide an indication that changes occur between supine and upright positions. Despite not demonstrating significant differences, it highlights the potential influence of position on the proximal sealing zone.

Observing EVAR patient in both supine and upright positions is important due to the suspicion that position-dependent endoleaks may occur more frequently in naturally occurring positions during daily life, such as the upright position. In previous upright studies, only the cross-sectional area of the aorta has been studied between upright and supine positions. These studies have reported no significant differences at multiple levels of the aorta [20, 21]. However, the potential effects or outcomes specifically associated with EVAR in an upright position have not been previously studied.

Although there were no notable position-dependent differences found in this study, the applied technique allows for aortic displacement analysis that could influence endograft sealing and apposition during an upright position. The two cases from this study with additional findings of less apposition around the aneurysm neck could potentially be causes of intermittent endoleaks [10]. However, upon retrospective evaluation, similar findings were observed in one patient that were initially documented as additional MRI findings but were not reported in the initial CTA report. This raises doubts regarding the added value of MRI in this specific patient, but it highlights that MRI is non-inferior to CTA in this instance. Furthermore, the study noted instances of endoleaks detected on CTA but not visible on MRI due to limitations in the field of view or the absence of direct visualisation of flow within the aneurysm. While 4D flow MRI has made it possible to utilise this technique for endoleak detection [22, 23], low-field MRI systems have only recently begun to overcome the main limitation of the field-related limited signal-to-noise ratio [24]. As the field of low-field MRI advances and increasingly incorporates modern gradient system and receiver array coils, as well as artificial intelligence architectures and algorithms, the benefits of visualising flow, as highlighted by 4D flow MRI, should be considered for inclusion in future follow-up studies [25].

While this study utilised MRA techniques for contrast-enhanced imaging in the abdominal aorta, as opposed to the conventional CTA, it is important to acknowledge some limitations associated with MRA. CTA has long served as the clinical standard for follow-up after EVAR, offering advantages in terms of cost-effectiveness, widespread availability, and ease of interpretation compared to MRA techniques [5]. However, it is crucial to emphasise that the objective of our current study is not to replace the established gold standard in the follow-up of EVAR patients. Instead, our aim was to determine if upright low-field MRI can provide additional information in the clinical management of patients with unexplained aneurysm enlargement.

A limitation of this feasibility study is its small sample size of only 10 patients, which precludes the demonstration of statistically significant differences. Nevertheless, the accuracy of the registration method in the aorta and endograft deformation objective fell above a clinically acceptable threshold. Additionally, the use of low-field MRI with rotating capabilities presents limitations in terms of image quality when compared to regular clinical MRI. While this study showed some cases to be non-inferior to CTA, the improvement of MRI hardware and imaging sequences has the potential to fully unlock the capabilities of upright MRI. Addressing scanner limitations such as being unable to use triggered scanning, optimising MRI pulse sequences, and incorporating state-of-the-art acquisition and reconstruction techniques could further enhance the potential of upright MRI as a complementary modality alongside CTA. Furthermore, the MRI coils used in this scanner are originally designed for musculoskeletal investigation [26], whereas dedicated coils for vascular imaging would yield better results. Another limitation of this study is the deviation from CTA, which has the benefit of directly visualising contrast in the aneurysm sac. In contrast, this study focused on assessing prominent vascular structures (*e.g.*, hypertrophic lumbar arteries) or indications for type Ia endoleak (*e.g.*, reduced apposition in the aneurysm neck). Our low-field MRI scanner could not achieve the high temporal resolution required to assess changes in signal intensity within the aneurysm sac. Besides, the addition of contrast agent could enhance flow visualisation in MRI, but this was not feasible in this study due to the MRI exams being conducted outside of a clinical setting. If similar clinical outcomes could be achieved, MRI could be considered a non-inferior alternative to standard CTA, as it avoids ionising radiation and potential contrast agent use [27].

In conclusion, a limited added clinical value of supine and/or upright low-field MRI in EVAR patients for endoleak detection has been demonstrated. The image quality of the tiltable MRI was confirmed to be inferior to that of CTA and varied among patients. In most cases, deformation between anatomical and endograft landmarks could be measured with an accuracy of approximately 6–7 mm. Considering this level of accuracy, deformation between the supine and upright positions was not detected in this study. However, if the scan quality is further improved, scanning in the upright position may provide added value for patients suspected of having a type Ia endoleak that cannot be verified with conventional CTA.

## Data Availability

All data generated or analysed during this study are included in this published article.

## Abbreviations

CDUS: Colour Doppler ultrasound
CTA: Computed tomography angiography
EVAR: Endovascular aneurysm repair
LRA: Left renal artery
MRA: Magnetic resonance angiography
MRI: Magnetic resonance imaging
MRI_0_: Supine MRI
MRI_81_: Upright MRI
RRA: Right renal artery
